# Assessment of the duration of continuous EEG monitoring in aneurysmal subarachnoid hemorrhage

**DOI:** 10.3389/fmed.2026.1922482

**Published:** 2026-10-14

**Authors:** Veronika Solnicky, J. Ricardo Carhuapoma, Jose I. Suarez, Robert D. Stevens, Eva K. Ritzl, Sarah E. Nelson

**Affiliations:** 1 The Permanente Medical Group, Department of Neurosurgery, Kaiser Sacramento Medical Center, Sacramento, CA, United States; 2 Departments of Neurology, Neurosurgery and Anesthesiology and Critical Care Medicine, Johns Hopkins University, Baltimore, MD, United States; 3 Department of Neurology, Hospital for Special Surgery, New York, NY, United States; 4 Department of Neurology, Johns Hopkins University, Baltimore, MD, United States; 5 Department of Neurology, Tufts Medical Center, Boston, MA, United States; 6 Departments of Neurology and Anesthesiology & Critical Care Medicine, Johns Hopkins University, Baltimore, MD, United States

**Keywords:** aneurysmal subarachnoid hemorrhage, delayed cerebral ischemia, electroencephalography, ictal-interictal continuum, length of stay, prognosis, seizures, vasospasm

## Abstract

**Background:**

Electroencephalography (EEG) can detect seizures and delayed cerebral ischemia (DCI) in patients with aneurysmal subarachnoid hemorrhage (aSAH). Because a standard duration of continuous EEG monitoring to capture these complications has not been established in aSAH, we compared a standard 6- or 7-day cEEG monitoring protocol versus longer periods of monitoring.

**Methods:**

We performed a retrospective analysis of aSAH patients ≥18 years old grouped by standard monitoring protocol (6–7 days, group 1) and those with longer monitoring (>7 days, group 2). Clinical characteristics, EEG features, length of stay (LOS) and functional outcome were compared using univariate analyses. Multivariable analyses tested the association between the groups and vasospasm, DCI, LOS, and modified Rankin Scale (mRS).

**Results:**

19 patients met criteria for group 1 and 16 patients for group 2. We found no difference in EEG's detection of worsening abnormal EEG activity, vasospasm and DCI. ICU and hospital LOS in group 2 were nearly double that of group 1 (ICU: 25.5 (19.5–35) vs. 14 (11–21) days, *p* = 0.0003; hospital: 32.5 (29.5–48) vs. 18 (13–29) days, *p* = 0.003). Good outcome (mRS ≤2) at discharge and at 6 months was similar in both groups (21.1% vs. 0% and 56.3% vs. 46.2%). In multivariable analysis, EEG duration was independently associated with ICU and hospital LOS but not with vasospasm, DCI, and mRS at 6 months.

**Conclusions:**

Longer EEG monitoring correlated with significantly longer ICU and hospital LOS but not with vasospasm, DCI, and functional outcome at 6 months. Larger studies to confirm our findings are warranted.

## 1 Introduction

Electroencephalography (EEG) remains an irreplaceable tool in the neurocritical care unit (NCCU). Seizures can be detected in up to a third of critically ill patients (1), and more than 90% can be non-convulsive and thus only detectable on EEG (2). In aneurysmal subarachnoid hemorrhage (aSAH), the number of patients estimated to be affected by seizures is 7%–18%, with non-convulsive status epilepticus occurring in approximately 3%–13% (3). In addition, EEG can be utilized in early detection of delayed cerebral ischemia (DCI; i.e., by assessing the alpha/delta ratio), before clinical symptoms develop and while secondary brain injury is preventable (4, 5), which is associated with increased morbidity and mortality in aSAH patients. Along with the benefit of being continuous and non-invasive, EEG is thus an important tool in caring for aSAH patients. Most recent guidelines agree that use of continuous EEG (cEEG) for seizure detection in patients with fluctuating neurological exam, depressed mentation, ruptured middle cerebral artery aneurysm, high-grade aSAH, intracerebral hemorrhage (ICH), hydrocephalus or cortical infarction is reasonable, in addition to use for monitoring for DCI in patients with high-grade aSAH (6).

What is less established is the optimal duration for EEG monitoring in these patients. While some centers routinely monitor aSAH patients for seizures and DCI, others likely only monitor these patients in response to clinical indications (3). Massachusetts General Hospital has reported monitoring all poor-grade aSAH patients for at least 10 days (7) while Johns Hopkins Hospital does so for 6–7 days (8). In a systematic review, cEEG duration in aSAH ranged anywhere from 2 to 16 days (3). While the cEEG monitoring duration at our center lies somewhere in the middle of this range, we sought to evaluate whether an extension of this duration or reconnection of the cEEG later in the patient's clinical course provided additional information that might impact clinical decision making, neurological outcome and length of stay using a cohort of consecutive aSAH patients.

## 2 Materials and methods

### 2.1 Patient selection

We performed a retrospective analysis of 49 aSAH patients admitted to the Johns Hopkins Hospital NCCU at our institution from March 2017 to April 2020 who underwent continuous EEG monitoring. Patients were ≥ 18 years old and had aSAH of any clinical and radiographical grade. Thirty-five patients completed at least 6 full days of consecutive cEEG monitoring (standard protocol for our ICU) and were included in further data analysis. Of note, at our institution, cEEG is used in conjunction with other tools (e.g., neurologic exam, CT, MRI, TCDs) to make clinical decisions. EEG day did not necessarily correspond to post bleed day; most EEGs were started on post bleed day 0–2, after aneurysm securement. Patients were categorized into two groups: Group 1 consisted of 19 patients who underwent the standard unit 6–7-day EEG monitoring protocol. Group 2 included 16 patients who underwent the standard protocol in addition to meeting one of the following criteria: cEEG was extended beyond the seven days (2 patients), EEG was re-connected for additional time during their hospital stay [either routine or continuous (12 patients)] or met both of these criteria (2 patients).

### 2.2 Standard protocol approvals, registrations, and patient consents

All procedures were performed in compliance with relevant laws and institutional guidelines and have been approved by the Johns Hopkins Institutional Review Board (April 2013; NA_00044076) with a consent waiver. Privacy rights have been observed.

### 2.3 Data collection

Demographic information (e.g., age and sex), clinical presentation [Hunt and Hess (HH) Scale, Glasgow Coma Scale (GCS), World Federation of Neurological Surgeons (WFNS) Scale], radiographic presentation (Modified Fisher Scale [mF]), hospital and ICU length of stay, occurrence and clinical suspicion for seizures and presence of vasospasm and DCI were abstracted from the electronic medical record. EEG features were obtained both from the electronic medical record and from EEG tracings acquired with Nihon Kohden (Tokyo, Japan) software and processed with software by Persyst (Solana Beach, CA, USA).

The principal outcomes of interest were the occurrence of seizures, vasospasm and DCI. Secondary outcomes were hospital and ICU length of stay as well as modified Rankin Scale (mRS) at discharge and at 6 months. mRS was obtained via telephone interview or abstracted from data available in the electronic medical record. Outcomes were dichotomized into favorable (mRS ≤2) and unfavorable (mRS >2).

### 2.4 EEG analysis

Data were extracted from the EEG reports created as part of routine clinical care. All clinical EEGs had been read by board certified epileptologists with experience in ICU EEG monitoring. The raw EEG was reviewed for select patients by VS and EKR (see below). In group 1 we reviewed the EEG report from the first (day 1) and last day (day 6 or 7) of the ICU EEG. In group 2, we also evaluated the EEG report from the first (day 1) and day 6 or 7 of the clinical protocol EEG duration. Additionally, for the two patients who had longer consecutive EEG monitoring, we also looked at day 8 and at the final day of continuous EEG monitoring (completed on day 8 and 13 for these two patients, respectively). For the 12 patients reconnected to EEG, we examined the first and last days of any additional continuous EEGs and also any routine EEGs. Finally, for the two patients who underwent both longer consecutive monitoring and additional EEGs, we looked at day 8 and the last day of the initial continuous EEG (day 9 for both patients), as well as a routine EEG and an additional day of continuous EEG monitoring, respectively.

We extracted the following raw EEG information: voltage, frequency, posterior basic rhythm, continuity, variability, reactivity, sleep, and asymmetry (Table 1). We also noted any epileptiform findings and ictal activity using American Clinical Neurophysiology Society (ACNS) Standardized Critical Care EEG Terminology (9) If information appeared to be missing, the raw EEG was reviewed by VS and EKR (VS is a board-certified epileptologist; EKR is a board-certified epileptologist who also passed the Critical Care EEG Monitoring Research Consortium Exam 1).

**Table 1:** EEG scoring system for EEG findings and EEG trend.

Category	Description	Score	EEG Background trend from EEG day 1 to day 6 or 7^a^	Score
Frequency	Theta-alpha/alpha-theta (normal)	0	For Frequency, Variability, Reactivity	
	Theta-delta (mild diffuse slowing)	1	Improving	0
	Delta-theta (moderate/prominent diffuse slowing)	2	Normal or Near Normal but Stable (Score 0/Score 1 or 2)	1
	Delta/burst suppression (severe/very abnormal diffuse slowing/suppression)	3	Abnormal but Stable (Score 3)	2
Variability	Normal/Good/Spontaneous	0	Worsening	3
	Fair (present but not normal)	1		
	Poor/Some/Minimal/Mild	2	For *PBR*	
	Absent	3	Improving	0
Reactivity	Good/Normal	0	Normal and Stable (Score 0)	1
	Atypical/Fair (present but not normal)	1	Abnormal but Stable (Score 1 or 2)	2
	Poor/Some/Minimal/Mild	2	Worsening	3
	Absent	3		
PBR	Present, normal	0	For *Continuity*	
	Present, abnormal (intermittent, asymmetric or slow)	1	Improving	0
	Absent	2	Normal or Near normal but Stable (Score 0/Score 1)	1
Continuity	Present	0	Abnormal but Stable (Score 2)	2
	Some discontinuity/mostly continuous	1	Worsening	3
	Absent/mostly discontinuous/burst suppression	2		
*Abnormal activity*	None	0	For *Abnormal activity*	
	Generalize slowing, GRDA, GPDs	1	Improving	0
	Focal slowing	2	Stable—without abnormal activity (Score 0)	1
	LPDs, LRDA, BIPDs	3	Stable—without clear epileptiform findings (Score 1 or 2)	2
	Spikes/sharp waves (focal)	4	Stable—with epileptiform findings/findings on the ictal-interictal spectrum (Score 3–5)	3
	Seizure	5	Worsening	4

PBR, posterior basic rhythm; GRDA, generalized rhythmic delta activity; GPDs, generalized periodic discharges; LPDs, lateralized periodic discharges; LRDA, lateralized rhythmic delta activity; BIPDs, bilateral independent periodic discharges.

a Group 1 Day 1 and 6 or 7; Group 2 last absolute EEG day compared to the last day of EEG prior to reconnect or day 6 or 7 of initial connection. Theta-alpha: predominantly theta (4 - < 8 Hz) with admixed alpha (8–12 Hz) frequency waveforms, alpha-theta: predominantly alpha with admixed theta. Theta-delta: predominantly theta with admixed delta (0 - < 4 Hz), delta-theta: predominantly delta with admixed theta, delta/burst-suppression: delta slowing without admixed faster frequencies or burst suppression pattern.

### 2.5 Assessment for vasospasm and DCI

Further, we looked at the presence of vasospasm and DCI. We observed the first date that vasospasm was documented in the electronic medical record by transcranial doppler (TCD), computed tomography angiography (CTA), magnetic resonance angiography (MRA), and/or digital subtraction angiography (DSA), and whether this occurred within the standard EEG monitoring protocol window; TCD reports noted normal upper limits of 70 cm/s for the MCA and 45 cm/s for the vertebral artery while no specific numeric cutoffs for vasospasm were noted in the reports of other modalities. We similarly observed when DCI occurred (or not) for each patient utilizing an established definition: “The occurrence of focal neurological impairment … or a decrease of at least 2 points on the Glasgow Coma Scale … This should last for at least 1 h, is not apparent immediately after aneurysm occlusion, and cannot be attributed to other causes by means of clinical assessment, CT or MRI scanning of the brain, and appropriate laboratory studies.” (10)

### 2.6 EEG scoring

In order to compare the EEG characteristics and pathology in a numeric fashion, a scoring system for EEG background (frequency, variability, reactivity, posterior basic rhythm (PBR), continuity, and abnormal activity) and EEG trend (from first to last day of EEG monitoring as defined respectively by the two patient groups) was developed by three board-certified neurologists (SEN, EKR, VS) **(**Table 1, additional data are in Supplementary Tables S1 and S2**)**. This scoring system was created solely for the organization and analysis of our data, is exploratory, and has not been validated in any other study. Mean and median trend background were calculated as follows: Mean: sum of trend scores for frequency, variability, reactivity, PBR, continuity divided by these five categories. Median: trend scores for frequency, variability, reactivity, PBR, continuity were ordered and the middle number selected. Early mean and median were similarly calculated, except that only the trend from day 1 to day 6 or 7 was used for both group 1 and group 2.

### 2.7 Statistical analysis

As this was a pilot study, we did not embark upon *a priori* sample size and power analysis. The sample size analyzed reflected one of convenience to include all patients admitted to our ICU with aSAH and undergoing cEEG. However, our sample size was approximately the number of “intracranial hemorrhage” patients in each arm of Rossetti's et al. study, which found no difference in mortality between cEEG- and repeated routine EEG-monitored critically ill patients with altered consciousness, although it did not differentiate types of intracranial hemorrhage (e.g., number with SAH) (11). Continuous variables were described as median (interquartile range) while categorical variables were noted as number (percentage). Univariate analyses were performed comparing demographics, clinical characteristics, EEG characteristics, and outcomes between patients in group 1 versus group 2 using Wilcoxon rank-sum tests for continuous variables; chi-square tests were used for categorical variables. Abnormal EEG activity trend was subdivided by severity into groups for analysis: (a) improving to stable without clear epileptiform findings vs. stable with epileptiform findings/findings on the ictal-interictal spectrum to worsening (0–2 vs. 3–4) as well as (b) improving to stable with epileptiform findings/findings on the ictal-interictal spectrum vs. worsening (0–3 vs. 4) **(**Table 1**).** Multivariable analyses were then completed examining the outcomes described above (e.g., development of vasospasm, mRS at 6 months, etc.) that included variables known to be associated with outcome in aSAH (e.g., age, clinical severity on admission such as HH or WFNS) in addition to EEG features (e.g., background trend, abnormal activity trend). We did not perform multivariable models with the outcome discharge mRS since EEG type (standard vs. longer monitoring) predicted the outcome perfectly in these analyses. In particular, logistic regression was utilized for binary outcomes (vasospasm, mRS, etc.) while linear regression was used for continuous outcomes (ICU length of stay, hospital length of stay). Missing data were not imputed. We did not adjust for multiple comparisons in our study as our findings were meant to be hypothesis-generating; we deemed our sample size also too small to warrant testing for interactions. All statistical analyses were performed using Stata 15.1 (StataCorp, College Station, TX). Statistical significance was set at *p* < 0.05.

### 2.8 Data availability

Our research data includes sensitive and protected information such as patient data and therefore is not suitable to be published or shared.

## 3 Results

A total of 49 patients with aSAH were admitted to the NCCU and placed on cEEG monitoring. Thirty-five patients completed the standard unit EEG monitoring protocol; 19 patients were monitored per protocol only (group 1), whereas an additional 16 patients (group 2) were monitored for an initial stretch of longer than 6–7 days, reconnected to EEG again (either routine or continuous) after the initial monitoring period, or a combination of the two. Fourteen patients did not complete monitoring as per the unit protocol (<6 days of monitoring) and were therefore excluded; there were no baseline differences between included and excluded patients (Supplementary Table S3). The protocol was not completed in these patients due to clinical decision making regarding ongoing need for continuous EEG monitoring; this was assessed in the context of individual patient needs and tolerance of the study, taking into account goals of care (3/14 patients), patient comfort (1/14 patients), additional surgical interventions (2/14 patients) and need for ongoing monitoring for further decision making (8/14 patients). Missing data included lack of mRS scores at 6 months for *n* = 6 patients (3 in each group), due to loss to follow up. EEG was applied and interpreted in a timely fashion, with all patients connected within a median (IQR) of two days of aneurysmal rupture [group 1: 2 days (1–3) vs. group 2: 1 day (1–3)] and within 1 day of aneurysmal securement [group 1: 1 day (1–2) vs. group 2: 1 day (0–2)] (Table 2).

**Table 2:** Univariate analyses comparing EEG groups 1 and 2^a^.

Variable	Group 1 (*n* = 19)	Group 2 (*n* = 16)	*P* value
Age (years)	61 (42–65)	58 (50.5–63.5)	0.77
Female Sex	15/19 (78.9%)	11/16 (68.8%)	0.49
Admission HH	3 (2–4)	3 (3–4)	0.77
Admission GCS	13 (8–14)	10.5 (8–14)	0.53
Admission mF	4 (3–4)	4 (3.5–4)	0.87
Admission WFNS	4 (2–5)	4 (2.5–4)	0.75
SAH onset to EEG Connection^b^	2 (1–3)	1 (1–3)	0.73
Trend Background Mean^c^	1.2 (0.6–1.6)	0.8 (0.6–1.6)	0.71
Trend Background Median^c^	1 (0–2)	1 (0.5–2)	0.67
Trend Background Early Mean^c^	1.2 (0.6–1.6)	1 (0.6–1.3)	0.71
Trend Background Early Median^c^	1 (0–2)	1 (1–1.5)	0.61
Worse Abnormal Activity Trend^d^	3/19 (15.8%)	6/16 (37.5%)	0.14
Worse Abnormal Activity Trend^e^	4/19 (21.1%)	6/16 (37.5%)	0.28
Seizures	1/19 (5.3%)	3/16 (18.8%)	0.21
Seizures while on EEG	1/19 (5.3%)	1/16 (6.3%)	0.90
Development of VSP	13/19 (68.4%)	13/16 (81.3%)	0.39
VSP while on EEG	11/13 (84.6%)	11/13 (84.6%)	1.00
Development of DCI	5/19 (26.3%)	6/16 (37.5%)	0.48
DCI while on EEG	1/5 (20.0%)	3/6 (50.0%)	0.30
ICU LOS	14 (11–21)	25.5 (19.5–35)	0.0003
Hospital LOS	18 (13 −29)	32.5 (29.5–48)	0.0003
mRS ≤2 at Discharge	4/19 (21.1%)	0/16 (0%)	0.051
mRS ≤2 at 6 Months	9/16 (56.3%)	6/13 (46.2%)	0.59

EEG, electroencephalography; HH, Hunt and Hess scale; GCS, Glasgow Coma Scale; mF, modified Fisher scale; WFNS, World Federation of Neurological Surgeons scale; ICU, intensive care unit; LOS, length of stay; VSP, vasospasm; DCI, delayed cerebral ischemia; mRS, modified Rankin Scale.

a Group 1 = standard EEG length. Group 2 = Longer EEG, EEG reconnected, longer EEG & reconnected. Data are listed as frequencies or as median (IQR).

b The term SAH onset is used here as aneurysms were not found on the initial digital subtraction angiography for 5 patients (2 in Group 1, 3 in Group 2).

c Using the scoring described in the manuscript.

d Grouped as better to poor vs worse.

e Grouped as better to intermediate vs poor/worse.

Age, sex, and admission characteristics including mF, HH, WFNS and GCS were comparable between the two groups (Table 2). ICU and hospital LOS for group 2 were nearly double that of group 1 (ICU: 25.5 (19.5–35) vs. 14 (11–21) days, *p* = 0.0003; hospital: 32.5 (29.5–48) vs. 18 (13–29) days, *p* = 0.003). Although numerically more patients in group 1 than in group 2 trended towards a good outcome (mRS ≤2) at discharge (21.1% vs. 0%), a similar proportion of patients in both groups achieved good outcome at 6 months (56.3% vs. 46.2%). Figure 1 visually describes the similarities and differences found between the two groups.

**Figure 1:**
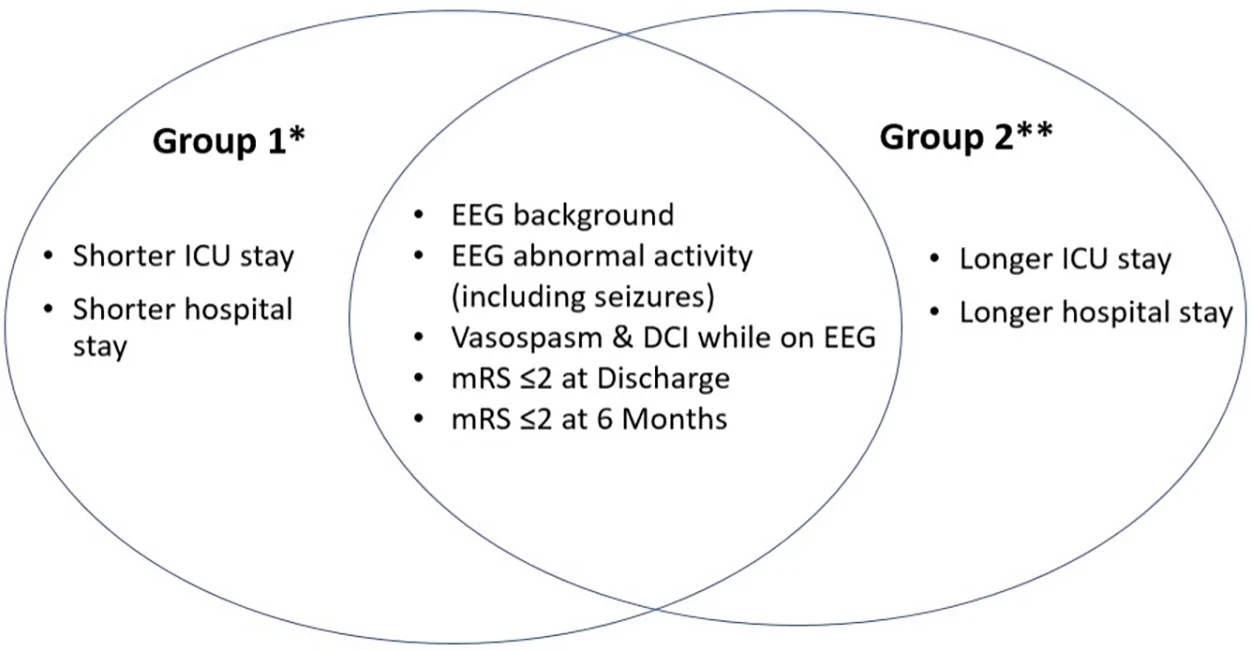
Comparison of characteristics between patients placed on EEG of different durations. *EEG duration per standard 6 or 7-day monitoring protocol. **EEG duration longer than standard protocol.

### 3.1 EEG and seizures

There was no difference in the proportion of patients with worsening abnormal EEG activity, characterized as increasing focal or epileptiform abnormalities from the first to the last day of the initial EEG recording period, between the groups (37.5% vs. 21.1%, *p* = 0.28). In our cohort, a total of 7 of 35 (20%) patients had witnessed or captured seizure activity: 3 patients presented to the hospital with clinical seizure-like activity (one in group 1 and two in group 2), 2 experienced witnessed seizure-like activity during the hospital stay (both in group 2), and 2 had confirmed non-convulsive seizures on EEG (one in each group) (Table 2). The most common reasons for continuing EEG monitoring beyond the standard unit protocol or reconnecting a patient were persistent change or worsening mental status and/or clinical concern for seizure (Supplementary Table S4).

### 3.2 Vasospasm and DCI

Vasospasm was found by TCD, CTA, MRA, or DSA in 26/35 (74.3%) patients. The proportion of patients who developed vasospasm in each group were similar (13/19 (68.4% vs. 13/16 (81.3%), *p* = 0.39). First detection of vasospasm of any severity occurred on post-bleed day (PBD) 0–14 for all patients (mean 4.3, median 3). Four patients (two from each group, 4/26 (15.4%)) developed vasospasm outside of the standard unit EEG monitoring protocol. Development of DCI at any point during the hospital stay, with majority occurring on PBD 3–15 (mean 9.1, median 9, one outlier at PBD 37), and occurrence of DCI while on EEG were similar in both groups.

### 3.3 Multivariable models

In multivariable analyses, we found that no variables in any models [e.g., age, clinical scores, EEG duration (i.e., being in group 1 vs. group 2), and EEG findings] were independently associated with the development of the outcomes vasospasm, DCI, or vasospasm and/or DCI. Further, neither EEG duration nor EEG findings were statistically significant in multivariable models for good outcome at 6 months. However, some clinical examination variables were independently associated with good outcome at 6 months: HH score remained significant when adjusting for age (OR 2.47 (95% CI 1.03–5.94), *p* = 0.042) and for age coupled with EEG duration (OR 2.46 (95% CI 1.03–5.92), *p* = 0.044). GCS was significant when adjusting for age (OR 0.77 (95% CI 0.60–0.97), *p* = 0.029) and also with EEG duration (OR 0.77 (95% CI 0.60–0.98), *p* = 0.034). WFNS score was also significant in similar models (OR 2.24 (95% CI 1.06–4.74), *p* = 0.035 and OR 2.22 (95% CI 1.05–4.72), *p* = 0.038, respectively). Longer EEG duration was independently associated with the outcomes ICU and hospital LOS (all *p* < 0.001), even in those models including clinical examination scores (which were not statistically significant in these models). No variables were independently associated with EEG duration. We were unable to successfully run multivariable models for good outcome at discharge likely given our small sample size, since it was found that EEG duration perfectly predicted the outcome in multivariable analyses.

## 4 Discussion

In this study, we found no difference in the abnormalities EEG was able to detect, including worsening abnormal activity (e.g., epileptiform abnormalities or seizures) and evidence of vasospasm and DCI in relation to duration of EEG monitoring between those aSAH patients monitored for standard unit 6–7 day EEG monitoring protocol vs. a longer duration (which included both extended EEG monitoring and/or reconnections). Further, we showed that EEG duration was independently associated with ICU and hospital LOS, and that there were no differences in functional outcome between the two EEG duration groups at discharge and at 6 months.

Consistent with prior literature, clinical examination scores (e.g., HH, GCS, WFNS) were independent predictors for good outcome at 6 months (6). We also noted a lower prevalence of captured electrographic seizures than has been noted in the literature (in their review, Kondziella et al. found that 7%–18% of aSAH patients had non-convulsive seizures and that 3%–13% had non-convulsive status epilepticus (3), which may in part have been due to our modest sample size or inclusion of low-grade aSAH patients compared to other studies (2, 3, 12, 13). Interestingly, of those patients reconnected or with longer EEG duration due to suspected clinical or non-convulsive seizures, no additional non-convulsive seizures were captured on EEG.

In addition, we found the majority of patients (22/26, 84.6%) who developed vasospasm were on EEG at the time they developed it. However, importantly, the same could not be said for DCI as only 4 of 11 patients (36.4%) with DCI were on EEG at time of development. Given the known capability of EEG to detect DCI (4–6), this suggests a possible benefit of longer EEG monitoring though the number of patients who developed DCI in our cohort was much smaller than the number who suffered from vasospasm.

Several studies have noted the value of EEG monitoring in the acute aSAH setting, but to our knowledge this is the first study to systematically analyze different EEG durations in order to help determine optimal EEG duration (and especially to capture events relevant to outcome) in this patient population. Contrary to some of the longer durations reported in the literature (3, 7), we found that shorter EEGs (i.e., one week) in general yield similar information as longer EEGs. Further, lengthier EEGs may delay a patient's discharge from the ICU and the hospital as we were able to demonstrate that EEG duration is independently associated with LOS. Nonetheless, long-term functional outcomes were similar in both groups. Our study largely supports the findings in Rossetti et al's trial comparing continuous versus routine EEGs, which demonstrated no difference in outcomes between continuous (30–48 h) and routine EEGs (two 20 min EEGs) in critically ill patients though seizures were better detected in patients who underwent continuous EEG; however, it is unclear how many patients in their study had aSAH (11).

One way that continuous EEG monitoring may have independently impacted LOS is through limitation in early mobilization, which has been shown to be beneficial in patients with aSAH (14). Although functional outcomes were comparable between our two study groups, mRS reflects only a single outcome measure and is unable to take into account additional implications of longer ICU and hospital stays such as quality of life measures, complications secondary to immobility (e.g., ICU acquired weakness, deep vein thromboses, increased ventilation days, delirium), increased costs and so on.

The cost-effectiveness of continuous EEG also needs to be considered when determining duration of monitoring (e.g., the probability of detecting a specific patient outcome (seizure, vasospasm, DCI) in a given period vs. cost of equipment and professional fees (technician and physician)). One study that evaluated EEG cost-effectiveness in children found that 1 h, 24 h and 48 h of EEG monitoring identified 55, 85% and 89% of seizing patients, respectively, suggesting that cost-effectiveness decreases as EEG duration increases (15). Similarly, Claassen et al. found that 88% of all patients who would eventually have seizures had their seizures noted on EEG within the first 24 h (2). Current costs of EEG in general (at least after-hours) can range from ∼$288 to ∼$818, with 48-hour 21 channel cEEG costing ∼$5,080 (16, 17), and seizures as a complication of aSAH can increase an aSAH patient's hospitalization costs by approximately $22,240 (18). No additional data are currently available regarding the cost-effectiveness of cEEG in aSAH patients specifically, for either seizure or DCI detection, and this is a potential topic for further studies.

We acknowledge that our study has several limitations. This was a retrospective, non-randomized, single center study and thus our results may lack generalizability and are subject to selection bias. Our sample size was modest, thus potentially limiting our ability to draw additional conclusions. Our results may also have been affected by indication bias in that the longer EEGs could have been performed due to clinical reasons and additional confounders not collected in this study thus leading to longer LOS, for instance; however, a comparison of patients with similar baseline clinical severities in the 2 EEG groups revealed no significant differences (Supplementary Table S5). Further, we acknowledge that reconnections for EEG may be performed for different clinical reasons than extending the duration of standard EEG monitoring so our data cannot necessarily be used to influence whether to increase the duration of monitoring for patients already on cEEG. We developed and utilized a new scoring system for our data analysis that is exploratory and has not been externally validated. We also did not have financial data in order to evaluate the cost-effectiveness of shorter vs. longer EEG monitoring. Another limitation is that vasospasm definitions can vary based on different detection modalities. It should also be noted that in some cases there was a time discrepancy of up to four days between aSAH onset and EEG initiation which could have affected comparisons between groups; nonetheless, most patients were started on EEG by post bleed day 2 (71.4%). Finally, it is possible that non-random missingness may have affected our results; however, missingness was minimal in our study and further there was an equal number of missing 6-month mRS values in each group. Nonetheless, strengths of our study included the numerous data points available for each patient, which permitted statistical analysis and tentative conclusions. The information in this study may be specifically of interest to those practicing in resource-limited settings where cEEG is not as readily accessible. Our results seem to warrant validation in larger, multi-institutional studies, particularly those that incorporate economic analysis or cost-effectiveness modeling.

## 5 Conclusion

In this hypothesis-generating study, shorter EEG duration seems to detect largely similar findings as longer EEGs, and lengthier EEGs may deter earlier patient discharge though similar long-term functional outcomes may still occur. Given the preliminary nature and limitations of this work as well as the potential clinical and financial ramifications of this topic, additional larger studies, especially in resource-limited settings, appear warranted.

## Data Availability

The datasets presented in this article are not readily available because our research data includes sensitive and protected information such as patient data and therefore is not suitable to be published or shared. Requests to access the datasets should be directed to solnicky.v@gmail.com.
