# Supplementary material for: Assessment of the duration of continuous EEG monitoring in aneurysmal subarachnoid hemorrhage
Source: Front Med (Lausanne). 2026 Oct 14;13:1922482. doi: 10.3389/fmed.2026.1922482 (PMC13713515; doi:10.3389/fmed.2026.1922482)
Supplement: Supplementary file 1 [file Supplementaryfile1.docx]

**SUPPLEMENTARY TABLES**

**Supplementary Table 1. Background Activity Scoring and Trend**

| **Patient #** | **EEG day** | **Frequency (score)** | **PBR**  **(score)** | **Continuity (score)** | **Variability**  **(score)** | **Reactivity**  **(score)** | **Mean** | **Median** |
| --- | --- | --- | --- | --- | --- | --- | --- | --- |
| **1** | 1 | Delta-theta (2) | Present (0) | Continuous (0) | Minimal (2) | Minimal (2) |  |  |
|  | 7 | Theta-delta (3) | Present (0) | Continuous (0) | Good (0) | Good (0) |  |  |
|  | Trend | Better (0) | Stable-Good (1) | Stable-Good (1) | Better (0) | Better (0) | 0.4 | 0 |
| **2** | 1 | Theta-delta (1) | Present, abnormal (1) | Continuous (0) | Some (2) | Some (2) |  |  |
|  | 7 | Theta-delta (1) | Present, abnormal (1) | Continuous (0) | Some (2) | Some (2) |  |  |
|  | Trend | Stable-Good (1) | Stable-Poor (2) | Stable-Good (1) | Stable-Poor (2) | Stable-Poor (2) | 1.6 | 2 |

**Supplementary Table 2. Abnormal Activity Scoring and Trend**

| **Patient #** | **EEG day** | **Abnormal Activity** | **Score** |
| --- | --- | --- | --- |
| **1** | 1 | None | 0 |
|  | 7 | None | 0 |
|  | Trend | Stable-without abnormal activity | 1 |
| **2** | 1 | Right temporal slowing | 2 |
|  | 7 | Right frontotemporal slowing | 2 |
|  | Trend | Stable- without clear epileptiform findings | 2 |

**Supplementary Table 3. Comparison of Included vs Excluded Patients**

| **Variable** | **Included (n=35)** | **Excluded (n=14)** | **P value** |
| --- | --- | --- | --- |
| Age (years) | 58 (47-65) | 66.5 (52-74) | 0.11 |
| Female Sex | 26/35 (74.3%) | 9/14 (64.3%) | 0.48 |
| Admission HH | 3 (3-4) | 4 (3-4) | 0.58 |
| Admission GCS | 12 (8-14) | 11.5 (7-14) | 0.96 |
| Admission mF | 4 (3-4) | 4 (3-4) | 0.91 |
| Admission WFNS | 4 (2-4) | 4 (2-5) | 0.73 |

Data are listed as frequencies or as median (IQR). Abbreviations: HH: Hunt and Hess scale, GCS: Glasgow Coma Scale, mF: modified Fisher scale, WFNS: World Federation of Neurological Surgeons scale

**Supplementary Table 4. Group 2 Indications for Extended EEG monitoring**

| **EEG Type** | **Patient #** | **Indication** |
| --- | --- | --- |
| Extended Monitoring | | |
|  | 1 | Extended due to a study protocol |
|  | 2 | Poor neurological exam |
| Reconnection | | |
|  | 1 | Sedated patient, limited exam |
|  | 2 | Decline in metal status |
|  | 3 | Clinical seizure vs anesthetic reaction post NIR vasospasm intervention |
|  | 4 | Fluctuating mental status |
|  | 5 | Altered mental status; clinical seizure (prompting 2^nd^ and 3^rd^ reconnection) |
|  | 6 | Decline in mental status, concern for seizure |
|  | 7 | Perseveration; fluctuating mental status |
|  | 8 | Change in mental status with decreased responsiveness |
|  | 9 | Fluctuating mental status concerning for non-convulsive seizure; reconnected due to a study protocol |
|  | 10 | Reconnected due to a study protocol |
|  | 11 | Altered mental status |
|  | 12 | Concern for seizure |
| Extended Monitoring & Reconnection | | |
|  | 1 | Extended due to a study protocol; reconnected due to concern for subclinical seizure |
|  | 2 | Poor mentation; reconnected due to ongoing altered mental status with concern for non-convulsive seizure |

**Supplementary Table 5. Comparison Between EEG Groups of Patients with HH 4-5 on Admission**

| **Variable** | **Group 1** | **Group 2** | **P value** |
| --- | --- | --- | --- |
| Discharge mRS | 5 (4-5) | 4.5 (4-5) | 0.85 |
| Discharge mRS ≤2 | 0/9 (0%) | 0/6 (0%) | N/A |
| 6 mo mRS | 5 (2-6) | 3 (2-6) | 0.66 |
| 6 mo mRS ≤2 | 2/7 (28.6%) | 2/6 (33.3%) | 0.85 |
| ICU LOS | 17 (13-22) | 25.5 (23-28) | 0.051 |
| Hospital LOS | 21 (18-34) | 32.5 (32-34) | 0.21 |
| VSP | 7/9 (77.8%) | 5/6 (83.3%) | 0.79 |
| DCI | 4/9 (44.4%) | 2/6 (33.3%) | 0.67 |

Abbreviations: HH: Hunt and Hess Scale, mRS: modified Rankin Scale, ICU: intensive care unit, LOS: length of stay, VSP: vasospasm, DCI: delayed cerebral ischemia.
